# Prognostic Significance of Dysregulated Epigenomic and Chromatin Modifiers in Cervical Cancer

**DOI:** 10.3390/cells10102665

**Published:** 2021-10-05

**Authors:** Aswathy Mary Paul, Madhavan Radhakrishna Pillai, Rakesh Kumar

**Affiliations:** 1 Cancer Research Program, Rajiv Gandhi Centre for Biotechnology, Trivandrum 695014, India; aswathym@rgcb.res.in; 2 Graduate Degree Program, Manipal Academy of Higher Education, Manipal 576104, India; 3 SAGENOME Private Limited, BioNest, Kochi 683503, India; 4 Cancer Research Institute, Swami Rama Himalayan University, Dehradun, Uttarakhand 248016, India; 5 Department of Medicine, Division of Haematology and Oncology, Rutgers New Jersey Medical School, Newark, NJ 07103, USA; 6 Department of Human and Molecular Genetics, Virginia Commonwealth University School of Medicine, Richmond, VA 23298, USA

**Keywords:** computational bioinformatics, cervical cancer, epigenomics, chromatin remodeling, fitness genes, new targets

## Abstract

To broaden the understanding of the epigenomic and chromatin regulation of cervical cancer, we examined the status and significance of a set of epigenomic and chromatin modifiers in cervical cancer using computational biology. We observed that 61 of 917 epigenomic and/or chromatin regulators are differentially upregulated in human cancer, including 25 upregulated in invasive squamous cell carcinomas and 29 in cervical intraepithelial neoplasia 3 (CIN3), of which 14 are upregulated in cervical intraepithelial neoplasia 2 (CIN2). Interestingly, 57 of such regulators are uniquely upregulated in cervical cancer, but not ovarian and endometrial cancers. The observed overexpression of 57 regulators was found to have a prognostic significance in cervical cancer. The collective overexpression of these regulators, as well as its subsets belonging to specific histone modifications and corresponding top ten positively co-overexpressed genes, correlated with reduced survival of patients with high expressions of the tested overexpressed regulators compared to cases with low expressions. Using cell-dependency datasets from human cervical cancer cells, we found that 20 out of 57 epigenomic and chromatin regulators studied here appeared to be essential genes, as the depletion of these genes was accompanied by the loss in cellular viability. In brief, the results presented here provide further insights into the role of epigenomic and chromatin regulators in the oncobiology of cervical cancer and broaden the list of new potential molecules of therapeutic importance.

## 1. Introduction

Cervical cancer is the fourth most common gynecological cancer in the global incidence and mortality rates [1]. Despite an effective HPV vaccination program, cervical cancer remains a threat in most developing countries. The focus of cervical cancer research has thus shifted to better understanding regulatory genomic insights of the disease in search of superior therapeutic options. The core of genomic regulation of gene expression is the upstream epigenomic regulatory mechanisms and chromatin remodeling processes, leading to overlapping and distinctive genomic features of regulatory molecules, further leading to molecular functions. In general, these modifying mechanisms could add or remove functional chemical marks such as acetylation, methylation, and phosphorylation groups to histones, and confer dynamic alterations in the ratio of active and repressed chromatin.

The progression of human cancer, at-large, to invasive phenotypes is driven by extracellular- and intracellular-signaling -dependent cellular pathways, including, activation of enzymatic activities by kinases [2,3,4]. Dysregulation of epigenomic and chromatin regulators has emerged as a major regulatory layer in cancer cells, which could potentially connect the external and internal signals to most, if not all, of the cancerous phenotypes. For example, DNA methylation and histone modifications are involved in the development and progression of cervical cancer at many levels [5]. EZH2-mediated histone H3K27me3 leads to DNMT3A downregulation, which in turn promotes the expression of genes such as HAVCR2 and LGALS9 to encode Tim3 and galectin-9 [6]. Tim-3 and galectin-9 confer immune tolerance to tumors upon overexpression in cervical cancer tissue compared to the adjacent normal tissues [6]. In addition to dysregulated expression of DNMT3A, DNMT1 also increases in low-grade cervical intraepithelial neoplasia (CIN) and squamous cell carcinoma (SCC) [7]. In general, DNA methylation patterns of HPV-positive and -negative cervical cells are distinct and correlate well with the levels of DMNT3A [8]. Papillomavirus-derived E7 oncoprotein was shown to thwart the immune response in an HPV-positive mouse model due to the hypermethylation of the chemokine CxCL14 promoter. Interestingly, CxCL14 can capture the attention of natural killer cells and CD8+ cells at the tumor site and thwart the immune response [9]. Another epigenomic molecule, polycomb repressive complex 2 (PCR2), downregulates the expression of E-cadherin when in conjunction with the histone deacetylase 1 (HDAC1), [10,11]. In addition to regulating the pathobiology of cervical cancer, promoter hypermethylation of Septin-9 (SEPT9) is a potential biomarker for early detection of the disease [12]. Another excellent example of the role of epigenomic regulators in supporting the contribution of E7 oncogene in evading the immune mechanism is the recruitment of HDAC to the promoter region of interferon regulatory factor 1 (IRF), thereby inhibiting the transactivation of IFN-β [13]. 

In addition to regulating the expression of target genes, epigenomic and chromatin regulatory complexes also contribute to the progression of cervical cancer. For example, a transcriptional inhibitory chromatin modification complex composed of the estrogen receptor alpha (ERα), HDAC1, JARID1B, and NF-kB transcription factor represses the expression of toll-like receptor 9 (TLR9) in the presence of E7 oncoprotein in cervical cancer cells [14]. This, in turn, leads to disrupted immune regulation. In addition, there are examples wherein HPV integration in cervical cancer cells was shown to be accompanied by increased expression and activity of apolipoprotein B MRNA editing enzyme catalytic subunit 3 (APOBEC3) [15]. Increased activity of APOBEC3 triggers mutations in the host genome through an abnormal DNA editing mechanism [16]. Another master epigenomic regulator, UHRF1, is overexpressed in cervical cancer cells and promotes proliferation by suppressing apoptosis [17]. In addition to cellular genes, there are examples of epigenomic regulations of viral oncogenes. For example, cellular TIP60 and P300 participate in the expression of HPV18 E6/E7 genes via the acetylation of a local control region (LCR) in cervical cancer cells [18]. 

In recent years, epigenomic and chromatin remodeling modifiers [19,20] have emerged as molecules of choice to modulate the responsiveness of cancer cells to specific therapeutics. Numerous molecules targeting HDACs and Sirtuin are undergoing attempted development as anticancer agents in different clinical trials for the treatment of cancers, including cervical cancer [21]. Many such molecules exert their antitumor activity by reducing the methylation of target genes and/or inhibiting the HDAC enzymes and restoring the acetylated chromatin in the vicinity of the target genes [21]. HDAC inhibitors exhibit antitumor activity in neuroblastoma [22]. As the growth of certain cancer types is driven by the formation of fusion proteins with epigenomic regulators, there are also reports to target such fusion proteins [23]. For example, translocation of bromodomain-containing protein 4 (BRD4) and formation of BRD4-NUTM1 fusion protein leads to a gain-of-function in the context of reading the histone acetylation in NUT midline carcinoma, and targeting BRD4 found effectively therapeutic in use [24]. Despite these examples, there are only a handful of examples of antitumor activity of epigenomic inhibitors in cervical cancer [25]. Therefore, to broaden the understanding of epigenomic regulation of cervical cancer, we examined the status and significance of a set of epigenomic and chromatin modifiers in cervical cancer. 

## 2. Materials and Methods

### 2.1. Curated Epigenomic Regulators

A list of epigenomic regulators was created from a public curation of epigenomic regulators from publicly available databases and literature. The public databases used included EpiFactors [26], dbEM [27], and CR2Cancer [28]. Collectively, we combined information about proteins modifying the histones, remodeling nucleosome, proteins modifying genetic material, and, in turn, affecting the expression of the gene, histone chaperone, histones, or histone variants. dbEM provides information on epigenomic regulators with roles in carcinogenesis, while CR2Cancer mainly focuses on the chromatin regulators. We removed the redundancy in epigenomic regulators and retained the epigenomic regulators with an approved gene symbol, corresponding functions. 

Epitranscriptomic landscape for cervical cancer. The cervical cancer dataset (GSE63514) [29] was analyzed to derive the epitranscriptomic landscape. The analysis performed comparing Normal (*n* = 24) vs. CIN1 (*n* = 14), Normal (*n* = 24) vs. CIN2 (*n* = 22), Normal (*n* = 24) vs. CIN3 (*n* = 40), and Normal (*n* = 24) vs. Cancerous (*n* = 28). Expression of certain epigenomic regulators was absent. As we could not find another dataset of similar classification and similar platform to Affymetrix U133A and Affymetrix U133 Plus 2.0, we only validated the result in another cancer sample, GSE7803 [30], where Normal samples (*n* = 10) were compared with squamous clear cell carcinoma (*n* = 21) and we validated the expression of the epigenomic regulators. 

Microarray data analysis was performed using R packages. For each group, the samples were loaded into R as CELL files, and samples were preprocessed [31]. The robust multichip average (RMA) [32] method was employed for the normalization of the samples. Expression values for each gene were then extracted using the exprs method and the differential expression analysis was performed using the limma [33] method between the two phenotypes for each study group. Genes with *p*-values less than 0.05 were removed from further analysis. About 20% of the differentially expressed genes could not map into proper HGNC symbols because of the lack of annotation. Later, we overlapped the differentially expressed epigenomic regulators from different cancer subtypes and performed further analysis. We also identified epigenomic regulators that are ubiquitously expressed despite the difference in cancer stage or cancer grade. 

The total differentially expressed 73 epigenomic gene set was later mapped against ovarian and endometrial cancers to verify the status of those cancer types. Pan-cancer-normalized TCGA RNAseq data were downloaded from the XENA browser for TCGA Ovarian Cancer (OV) (*n* = 308) and TCGA Endometrioid Cancer (UCEC) (*n* = 201) [34]. To derive the status of 73 epigenomic regulators in these two cancer types, only expression profiles for epigenomic regulators were curated for the above-mentioned cancer types. For each cancer type, epigenomic regulators were classified into upregulated or downregulated based on the average expression across samples. Following classification, the epigenomic regulators were overlapped and validated the expression status. We removed the genes that are expressed in ovarian or endometrial cancer from our gene set and then performed functional classification of the final gene set to identify major dysregulated functional groups. The expression epigenomic regulator was also cross-referenced with the TCGA cervical cancer dataset [35,36]. 

### 2.2. Enrichment and Correlation Analysis

Two separate enrichment analyses were performed. First, we took the 57 gene test dataset and performed gene regulatory network analysis using Network Analyst [37]. The gene test dataset was searched against the Signor database [38]. A direct graph represented each relationship between genes. Each signaling between the genes was associated with an effect. Next, we shortlisted the top 4 upregulated genes from the final gene set and took them for correlation analysis. The correlated gene information was collected from the cBioPortal database. Later, we constructed a network using the top 4 upregulated genes and corresponding correlated genes having a correlation value greater than 0.4 using Cytoscape-version 3.8 [39]. The obtained cluster was subjected to functional analysis using ClueGO and CluePedia [40,41]. 

### 2.3. Prediction of Interaction among Cervical Focus Gene Set Its Functional Annotations

Genes/proteins create changes in the biology of the cells based on their interaction with other molecules. We therefore decided to better understand the role of epigenomic regulators by investigating protein–protein (PPI) interactions. These epigenomic regulators from the microarray results were subjected to string analysis [42]. Protein–protein interaction analysis was performed separately for each major functional classification, such as histone phosphorylation, other histone modifications, and chromatin remolding complex. Interaction between the genes (proteins) is visualized in the form of a network. Each protein we entered was represented as nodes and their connection as edges. The connections/edges between the proteins are of different widths, indicating different evidence of an interaction. The line indicates the existence of fusion, evidence for the existence of neighborhood, co-occurrence of proteins, experimental evidence of protein, interaction evidence curated from text mining, and interaction evidence from the database, while the black line indicates the existence of co-expression. We identified protein–protein interaction as a different category as this can indicate the connection between phenotype and the epigenomic regulator expression. 

### 2.4. Prognostic Validation of Cervical Cancer Focus Set and Shared Gynecological Genes

SurvExpress, a web-based platform, was used to predict the prognostic possibility of epigenomic regulators for cervical cancer [43]. Only one dataset was available under the cancer type, selected cervical cancer. Hence, we selected CESC-TCGA cervical squamous cell carcinoma and endocervical adenocarcinoma in July 2016. The dataset contains 191 samples. Survival analyses of epigenomic regulators for each major dysregulated functional group were conducted separately. After entering the gene set, the symbols were mapped against the SurvExpress database. All the gene symbols were found to be mapped. The data were censored based on survival days and dividing the data into two risk groups: high and low risk. 

### 2.5. Fitness Dependency Analysis of Epigenomic Regulators 

The fitness score for 57 cervical-cancer-specific epigenomic regulators was curated from a CRISPR-Cas9-mediated knock-out study in 14 cervical cancer cell lines from the project score database [44]. We analyzed the functional loss of cell lines after the knock-down based on the score. The fitness score for each gene was plotted using R studio and classified the genes as essential and non-essential. 

## 3. Results and Discussion

### Epitranscriptomic Landscape of Cervical Cancer 

We first curated 917 epigenomic regulators and chromatin modifiers with roles in DNA methylation, histone methylation, acetylation, phosphorylation, ubiquitination, histone variants, transcription factors, and chromatin remodeling regulatory steps (Table S1, Figure S1a). About 85% of curated molecules retained the functional information from the database or literature, while 117 molecules had no defined functions. This also included 93 molecules with roles in multiple cellular processes, including histone acetylation as the largest functional group.

**Figure 1:**
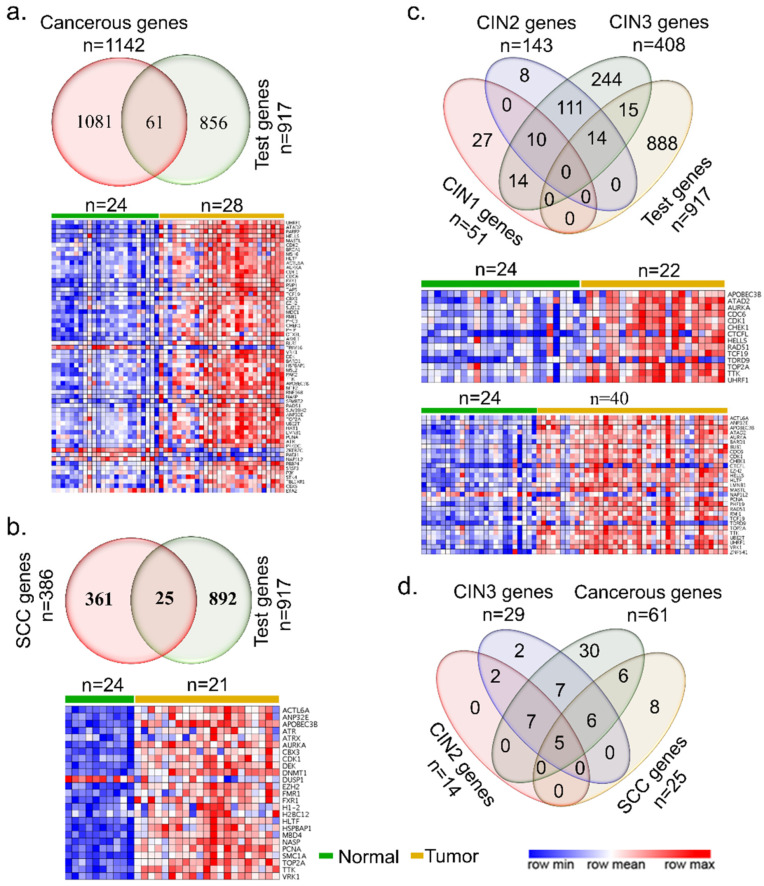
Epigenomic and chromatin regulators in cervical cancer. The Venn diagrams show overlap among the epigenomic and chromatin regulators, and expression heatmaps between the normal and cancerous genes (**a**), squamous cell carcinoma (**b**), CINs (**c**), and overlap under different cancerous conditions (**d**).

To understand the general significance of epigenomic modifiers in cervical cancer, we used a cancer gene dataset to assess the status of epigenomic modifiers as cancer-associated genes. We found 61 of the epigenomic modifiers to be cancer genes, and these were distinctively upregulated in cervical cancer specimens compared to non-cancerous adjacent normal tissue (Figure 1a). Of the 61 genes, 5 were downregulated, while others were upregulated (Table S2). Interestingly, 25 epigenomic and chromatin modifiers were differentially expressed in invasive squamous cell carcinoma tissue (Figure 1b, Table S3). Next, we determined the status of differentially expressed genes (*p*-value < 0.05) in cervical intraepithelial neoplasia (CIN)-1, -2, and -3, and found that 29 epigenomic modifiers were differentially expressed in CIN3, of which 14 were shared in CIN2 (Figure 1c, Table S4). Interestingly, all 14 differentially expressed genes shared between CIN2 and CIN3 were upregulated. Only one gene (i.e., nucleosome assembly protein 1 like 2 (NAP1L2), [45]) was downregulated in CIN3. Further overlapping of differentially expressed epigenomic modifiers among CIN2, CIN3, SCC, and cancerous genes revealed a general overlap of molecules among all cervical cancer sub-types (Figure 1d). 

**Figure 2:**
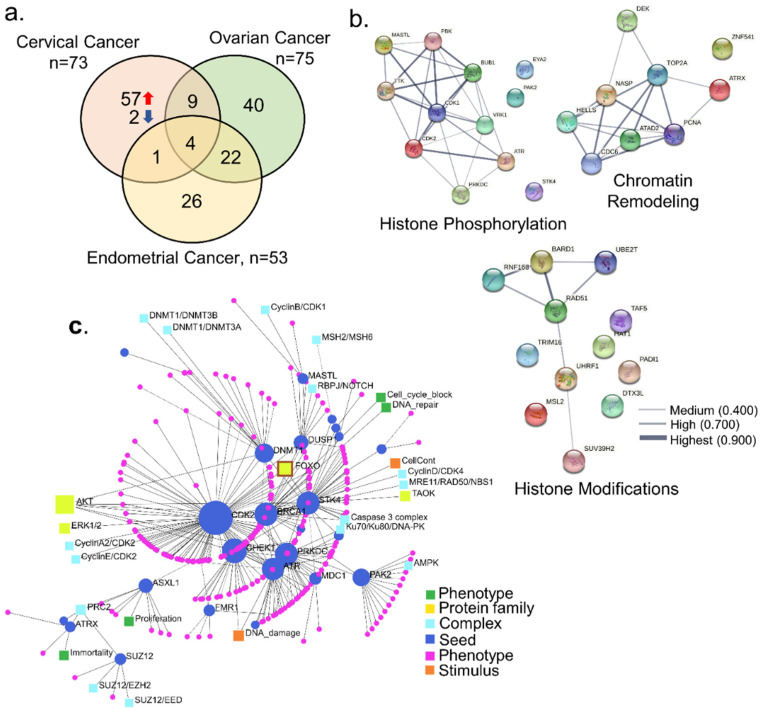
Significance of cervical-cancer-specific epigenomic and chromatin regulators. (**a**) Venn diagram representing the intersection of differentially expressed epigenomic regulators in cervical cancer with ovarian and endometrial cancer. (**b**) Protein–protein interaction of functional clusters; the color of the edge represents the strength of interaction. (**c**) The concentric circle image represents signaling enrichment of 57 epigenomic and chromatin regulators.

To assess the generality of the noticed dysregulation of 73 dysregulated epigenomic regulators in cervical cancer, we examined the expression status of these genes in ovarian and endometrial cancers (Figure 2a). We found that 57 epigenomic modifiers are uniquely dysregulated in cervical cancer (Table S5). Among these 57 genes, the largest functional group was of molecules with a role in histone phosphorylation (*n* = 12), followed by other histone modifications (*n* = 12) and chromatin modifiers (*n* = 9) (Figure S1b). Interestingly, we found evidence of protein–protein interactions within each of these three classes of differentially expressed epigenomic modifiers in cervical cancer (Figure 2b), implying that many of these molecules might work and/or converge onto the same set of functions. Signaling network enrichment analysis revealed seed molecules, complexes formed, protein families, stimulus, and phenotypes. Genes such as CDK2, CHEK1, BRCA1, PRKDC, STK4, ATR, DNMT1, PAK2, DUSP1, and ASXL1 were identified as the seed molecules. The analysis also identified the proliferation, DNA repair, immortality, and cell cycle as potential phenotypic effects caused by the alterations in the shortlisted genes.

**Figure 3:**
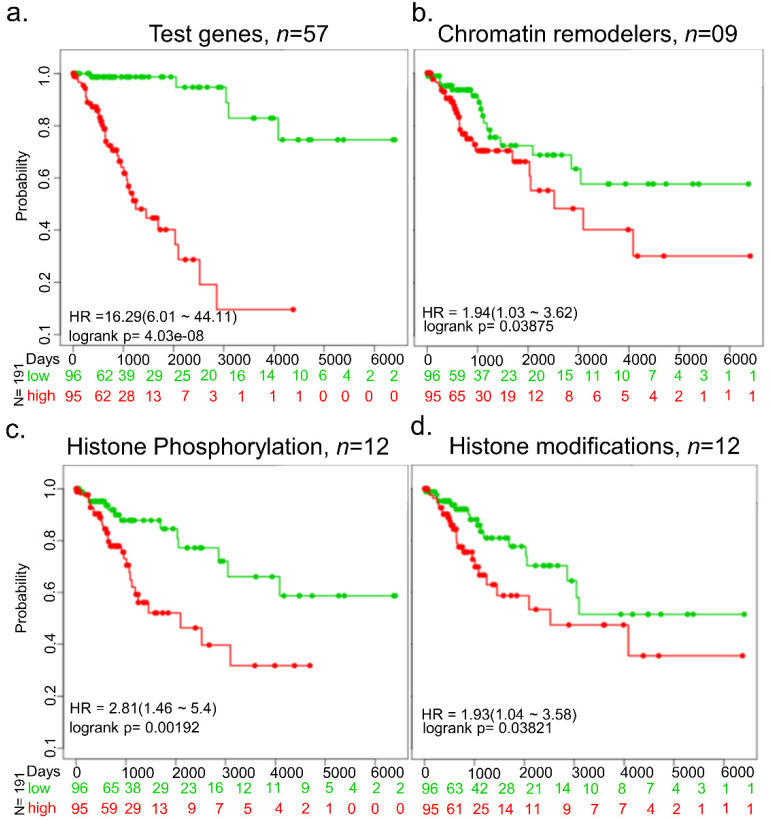
Survival analysis of cervical cancer-specific epigenomic and chromatin genes. (**a**) Analysis using 57 epigenomic and chromatin modifiers genes. (**b**) Analysis using 9 chromatin remodeler genes. (**c**) Analysis using 12 histone phsphorylation genes. (**d**) Analysis using 12 histone modification genes. Numbers below the X-axis represent the number of patients not facing an event for a long time for each group. N represents the number of total cervical cancer samples.

We next assessed the prognostic significance of the 57 upregulated epigenomic or chromatin modifiers in cervical cancer and noticed a clear distinction of the survival duration of patients expressing high versus low expressions of these modifiers (Figure 3a). Further, we determined the prognostic significance of the above upregulated molecules with a role in histone phosphorylation, histone modifications, or chromatin modification functional classes (Figure 3b–d). Like the collective analysis of 57 upregulated molecules, we found that molecules belonging to these functional groups also showed a positive correlation between the duration of survival and increased levels of expression of molecules within each functional group. 

**Figure 4:**
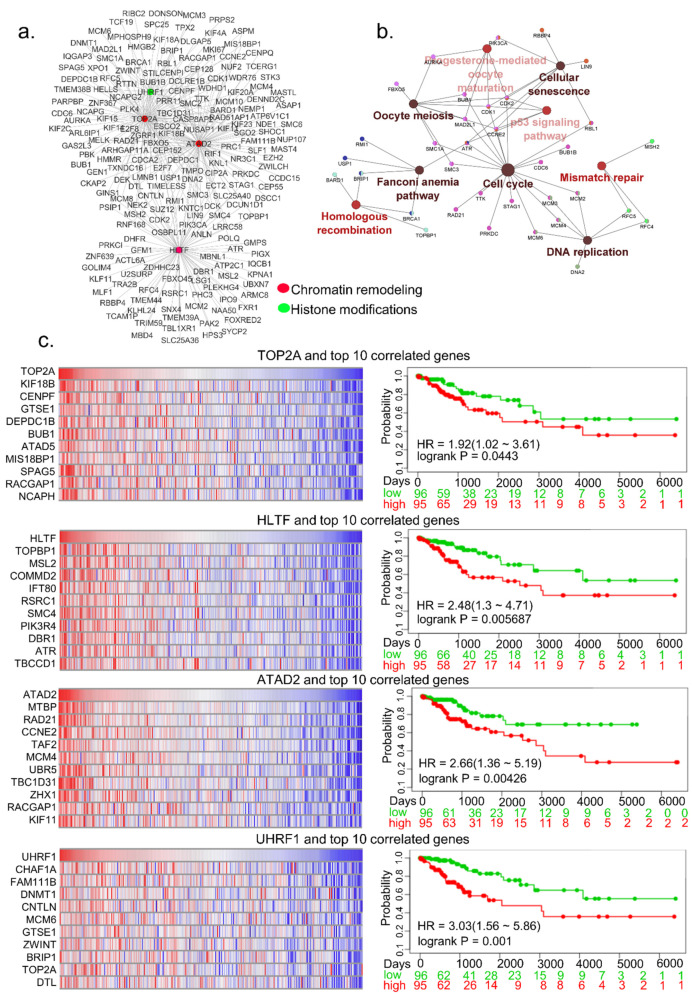
Significance of highly upregulated epigenomic and chromatin regulators in cervical cancer. (**a**) Network of four epigenomic and/or chromatin modifiers, upregulated over 2-fold, and its correlated genes. Epigenomic regulators are represented with colored dots. (**b**) KEGG pathway enrichment analysis of epigenomic regulator and its correlated genes. Larger nodes, the enriched pathway, and smaller nodes represent the genes involved in the pathway. (**c**) Heatmap representation of mRNA expression of epigenomic regulator and top 10 correlated genes (right panel), and Kaplan–Meier curves of four top upregulated epigenomic regulators and their correlated genes in CESC-TCGA cervical squamous cell carcinoma. Red and green color represents high and low risk, respectively. The X-axis represents survival days. Numbers below the axis represent the number of patients not facing an event along time for each group.

To further understand the relationship between the noticed upregulated regulators of epigenomic and chromatin modification, we selected four highly upregulated genes, two-fold or more, for network analysis, and their correlated genes for network analysis (Figure 4a). The potentially enriched KEGG pathways of top four upregulated epigenomic regulators and their correlated genes include both proliferative and genomic alteration pathways such as cell cycle, cellular senescence, DNA replication, p53 signaling pathway, mismatch repair, and homologous recombination (Figure 4b). To determine the relevance of the correlated genes in the context of the four selected functional classes of epigenomic modifiers, the data in Figure 4c illustrate the expression of such genes as heatmaps. To assess the significance of the levels of expression of these epigenomic and chromatin regulators and their top 10 positively correlated genes, we performed a survival analysis of cervical cancer patients from who these datasets were generated. We found that over-expression of co-expressed genes correlated well with shorter survival of patients compared to patients with low expression of these genes (Figure 4c, right panel). In brief, these observations suggested that many of the observed upregulated epigenomic and chromatin modifiers in cervical cancer may contribute to poor prognosis in conjunction with co-overexpressed cellular genes. 

**Figure 5:**
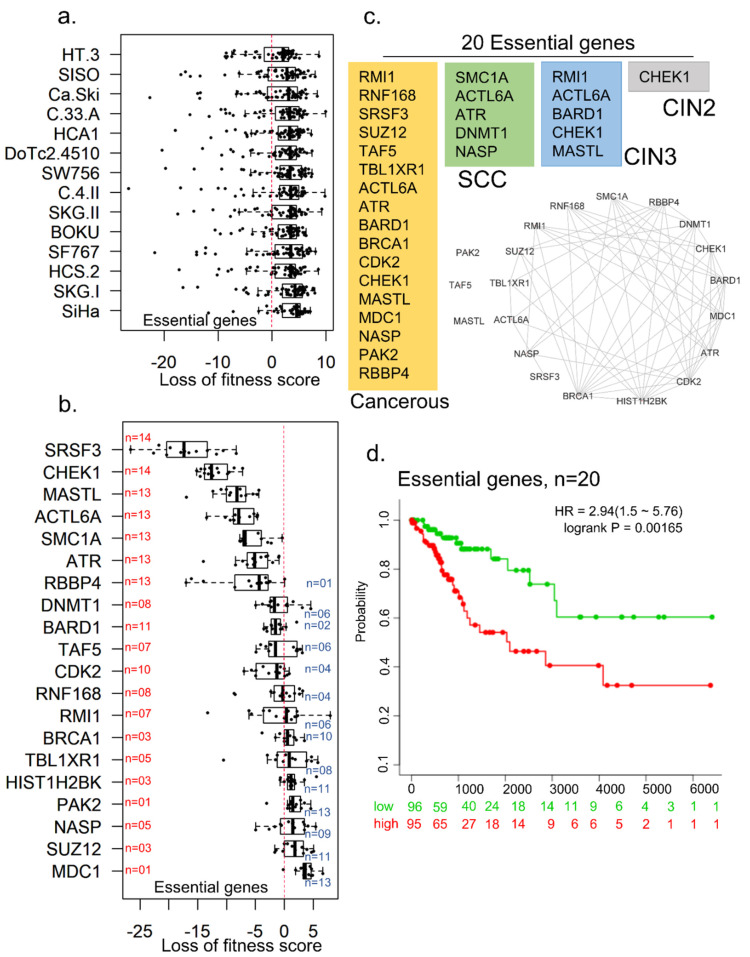
Role of epigenomic and chromatin regulators as fitness targets. (**a**) Fitness dependency distribution of 57 test regulators in cervical cancer cell lines. (**b**) Twenty essential genes cause loss of function on depletion in cervical cancer cell lines. The red and blue numbers represent the number of dependent and not dependent cell lines for each gene, respectively. (**c**) Protein–protein interaction network and expression classification of 20 essential epigenomic regulators. (**d**) Kaplan–Meier curves for 20 essential genes. Red and green color represents high and low-risk patients, respectively (*n* = 191). The X-axis represents survival days. Numbers below the axis represent the number of patients not facing an event over time for each group.

To understand the role of 57 differentially upregulated epigenomic modifiers molecules in cervical cancer cells’ viability, we assessed the fitness dependency of these molecules using a recently developed cell-dependency map of cancer genes [46,47,48]. The cancer gene dependency dataset involved cell viability data from CRISPR-Cas9-mediated depletion of about 7460 genes in well-characterized cell lines, including cervical cancer cell lines. We focused on a set of cervical cancer cell lines: Ca-Ski, HCS-2, HT-3, DoTc2-4510, C-4-II, C-33-A, BOKU, SISO, HCA1, SKG-II, SKG-I, SW756, SF767, and SiHa, as the cell models to assess our hypothesis (Figure 5a). Interestingly, the cell-dependency dataset contains fitness values of 55 out of 57 test molecules in cervical cancer cell lines (Table S6). We found that 20 of 57 epigenomic and chromatin regulators appear to be essential for the cellular fitness of cervical cancer cell lines; knocking down these genes affects the viability of cells, raising the possibility of developing some of these molecules as therapeutic targets. Examples of essential cell fitness genes include SRSF3, CHEK1, MASTL, ACTL6, SMC1A, ATR, and RBBP4 (Figure 5b). Interestingly, we found a higher rate of interconnection among these 20 fitness/essential genes (Figure 5c). About 75% of these molecules possess the property of heterocyclic compound binding, which targets anticancer drugs [49]. The cancerous relevance of these genes was evident by the observation that 17 essential genes were dysregulated at the cancerous stage. To assess the potential importance of these 20 genes, we performed a multivariant analysis of genes in the overall survival of patients with cervical cancer (Figure 5d). We found that collective overexpression of the noticed 20 essential genes correlates well with shorter survival of patients than patients with lower expression. 

In brief, our analysis identified molecules commonly dysregulated in most sub-types of cervical cancer, raising the possibility of shared epigenomic mechanisms underlying the progression and invasion of distinct cervical cancer sub-types. Interestingly, we failed to notice a dysregulated expression pattern of epigenomic regulators in the earliest recognizable pathologic lesions in the cervical tumorigenesis spectrum, namely CIN1, as opposed to progressive dysregulation in lesions including CIN2 to CIN3. This may imply that dysregulated expression of epigenomic and chromatin regulators could be preferentially involved in cancer progression rather than in the initiation of cervical cancer as judged by our finding in CIN1. Our bio-informatics findings provide a set of new epigenomic and chromatin regulators of cervical cancer for subsequent validation in appropriate cellular models and raise new questions about the mechanisms of regulation and functional significance of the noticed upregulation of molecules that might be unique to cervical cancer.

## Data Availability

The data presented in this study are available in the Supplementary Material.

## Supplementary Materials

The following are available online at https://www.mdpi.com/article/10.3390/cells10102665/s1, Figure S1: Functional classification of curated epigenomic regulator list; Figure S2: Distribution of 35 genes’ fitness dependency scores in cervical cancer cell lines that are not essential for the viability of the cell; Table S1: List of epigenomic regulators with its functional annotation; Table S2: Differentially expressed epigenomic regulators in cancerous cervical cancer dataset; Table S3: Differentially expressed epigenomic regulators in invasive squamous cell carcinoma; Table S4: Differentially expressed epigenomic regulators in CIN-2/-3 cervical cancer dataset; Table S5: 57 unique epigenomic regulars for cervical cancer when compared to Ovarian and endometrial cancer; Table S6: Fitness score for 55 epigenomic regulators in different cervical cancer cell lines.
