# Supplementary material for: Prognostic Significance of Dysregulated Epigenomic and Chromatin Modifiers in Cervical Cancer
Source: Cells. 2021 Oct 5;10(10):2665. doi: 10.3390/cells10102665 (PMC8534148; doi:10.3390/cells10102665)
Supplement: Supplementary file 1 [file cells-10-02665-s001.zip › Supplimentary_figures.pdf]

a.

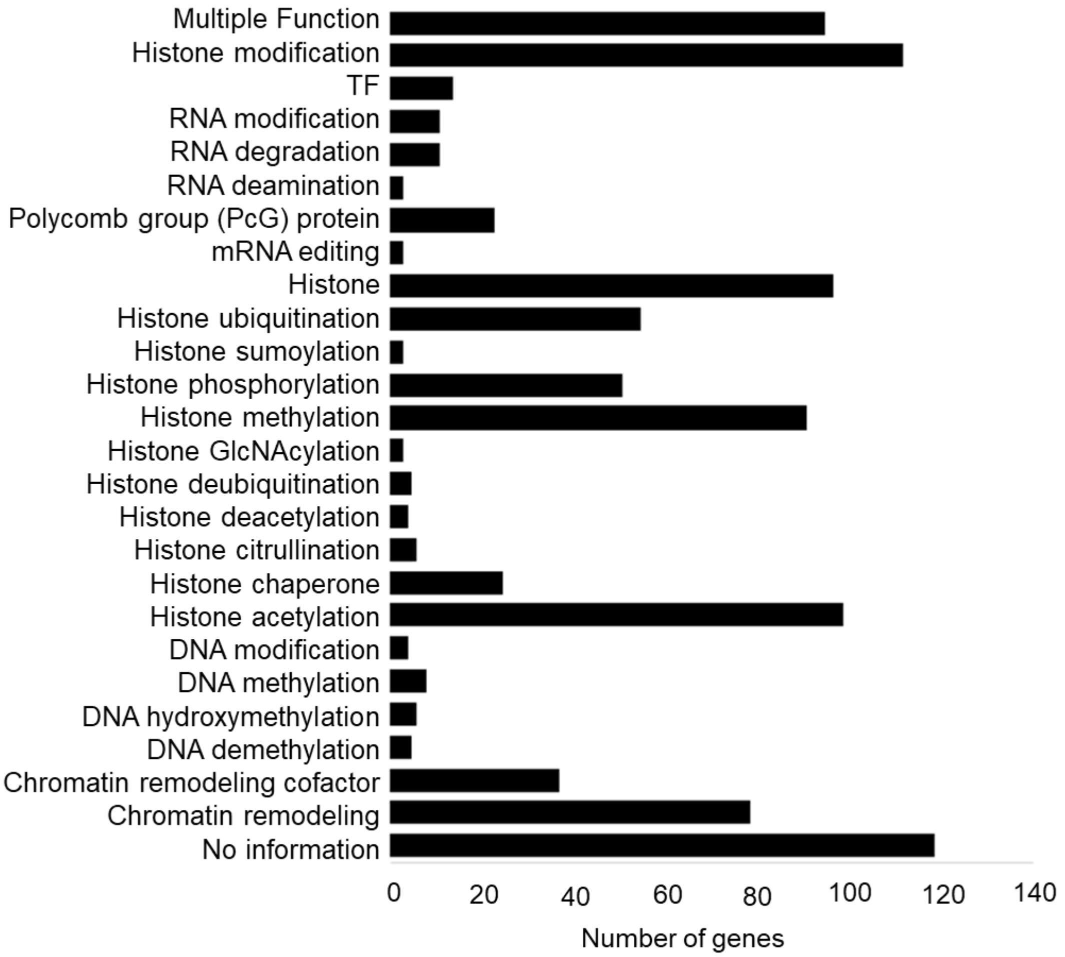

b.

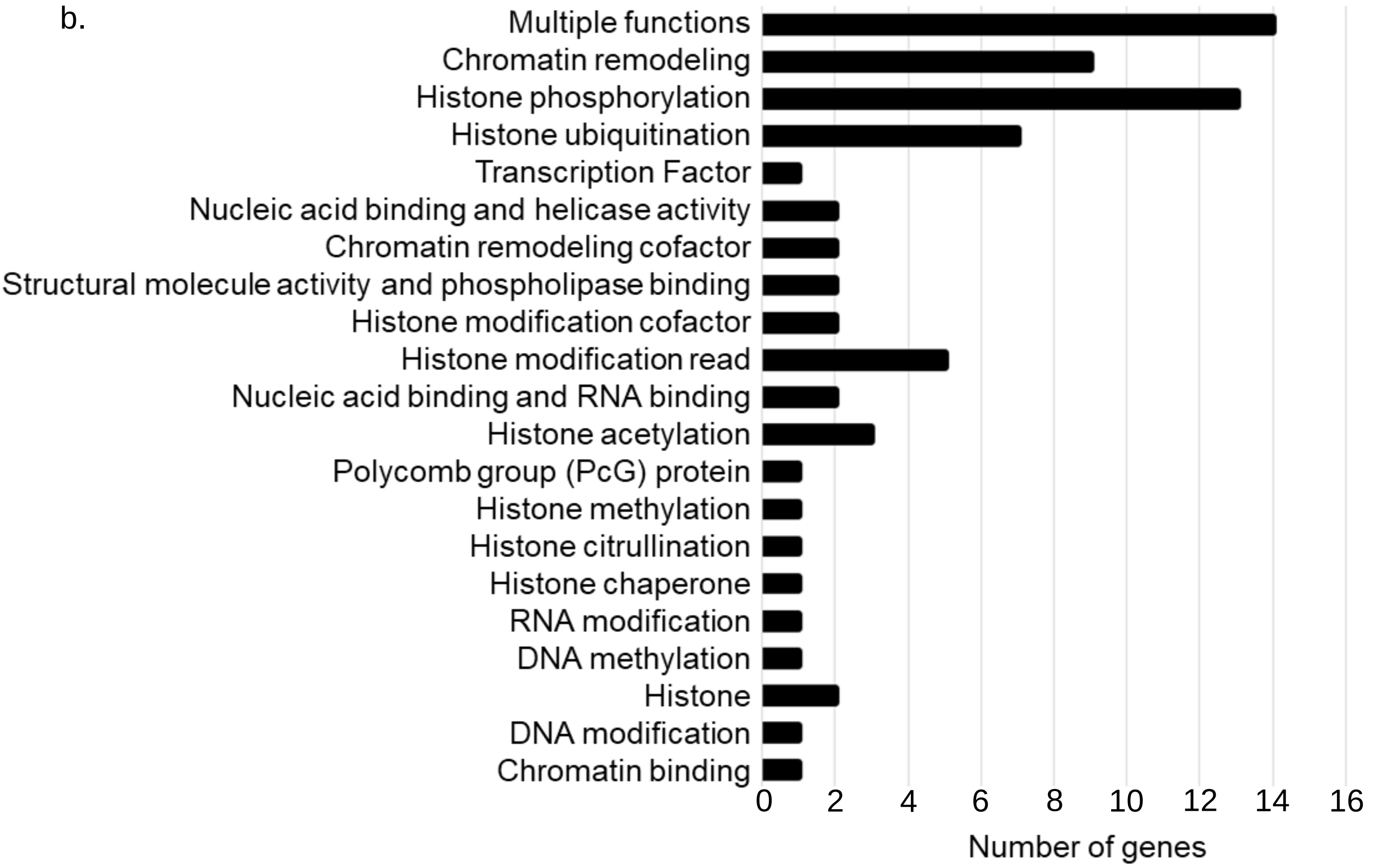

Supplementary figure S1: Epigenenomic and chromatin remodeling regulators. a, Classes of molecules curated in the present study. Y-axis represents the functional classification and X-axis represents the number of genes in each functional group, and b, Functional sub-types of 73 differentially expressed epigenomic and chromatin regulators.

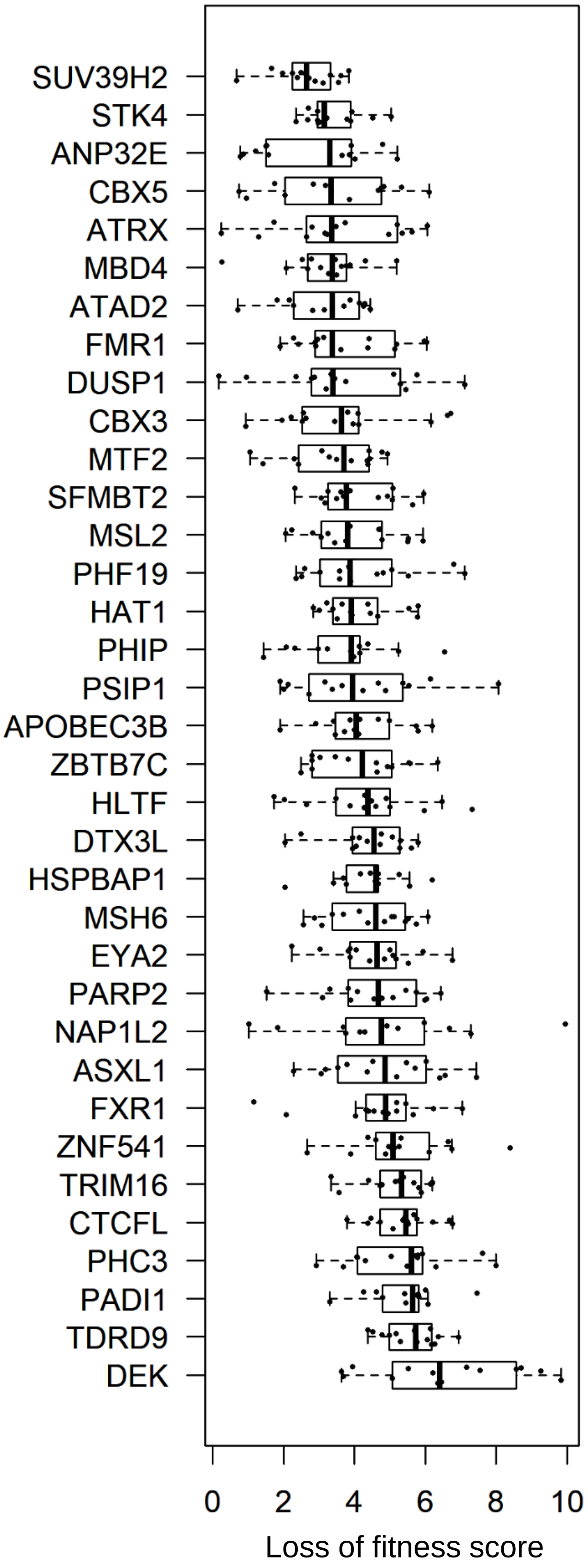

Supplementary figure S2: Genes with poor fitness dependency values in cervical cancer cell lines.
